# Neuroendocrine Carcinoma at the Sphenoid Sinus Misdiagnosed as an Olfactory Neuroblastoma and Resected Using High-Flow Bypass

**DOI:** 10.3390/diagnostics12071674

**Published:** 2022-07-09

**Authors:** Kosuke Takabayashi, Takafumi Shindo, Tomoki Kikuchi, Katsumi Takizawa

**Affiliations:** 1 Department of Otorhinolaryngology, Japanese Red Cross Asahikawa Hospital, Asahikawa 070-8530, Japan; kosuketakabayashi@gmail.com; 2 Department of Neurosurgery, Japanese Red Cross Asahikawa Hospital, Asahikawa 070-8530, Japan; ktaki@asahikawa-rch.gr.jp; 3 Department of Pathology, Japanese Red Cross Asahikawa Hospital, Asahikawa 070-8530, Japan; kt103296@tsc.u-tokai.ac.jp

**Keywords:** skull base, high-flow bypass, sustentacular cell, endoscopic sinus surgery, internal carotid artery

## Abstract

In the diagnosis of olfactory neuroblastoma (ONB), the presence of S-100–positive sustentacular cells surrounding the tumor is important; however, these are also present in normal nasal sinus epithelium. Although ONB often has a different final diagnosis, complete resection of the tumor has a good prognosis and minimally affects the patient’s treatment plan. When the tumor extends around the internal carotid artery (ICA), complete resection is difficult due to the high risk of vascular injury; revascularization using high-flow bypass can avoid this complication. In the present case, the tumor was located in the left sphenoid sinus and extended around the ICA. Preoperative biopsy tissue was positive for neuroendocrine markers and slightly positive for S-100 protein, leading to a diagnosis of ectopic ONB. High-flow bypass revascularization with trapping of the ICA allowed complete tumor resection. The postoperative histopathological diagnosis was neuroendocrine carcinoma, showing no S-100 protein-positive cells. There was no sign of recurrence at 30 months after surgery without additional treatment. This case demonstrates that the presence of S-100 protein-positive cells in ONB may be misleading. Although misdiagnosis of ectopic ONB should be anticipated, a complete resection of the tumor is an effective treatment strategy.

## 1. Introduction

Cases with a preoperative diagnosis of olfactory neuroblastoma (ONB) should consider the possibility of a different postoperative diagnosis [1,2]. The imaging features of ONB are nonspecific; thus, diagnosis using imaging modalities alone is difficult and warrants histopathology [1]. Misdiagnosis has a negative effect on patient prognosis, and immunohistological staining is essential for accurate diagnosis [1,2,3].

Malignant tumors extending around the internal carotid artery (ICA) are difficult to resect completely because of the risk of ICA injury. High-flow bypass allows for complete removal of malignant tumors that extend around the ICA [4,5,6].

We report the case of a patient who was diagnosed preoperatively with ectopic ONB of the left sphenoid sinus extending around the ICA; however, the postoperative diagnosis was neuroendocrine carcinoma (NEC). Complete removal of the tumor was performed using high-flow bypass to resect the ICA concomitantly with the tumor. Complete resection of the tumor with high-flow bypass is a treatment strategy worth considering given the low diagnostic accuracy of skull base malignancies. In particular, we describe in detail how both the pathologists and clinicians misdiagnosed the tumor as ONB based on the preoperative biopsy findings. We would like to emphasize that there is a risk of misdiagnosis in situations where the diagnosis is clinically convincing, even if immunohistological staining shows atypical findings.

## 2. Case Report

**Figure 1:**
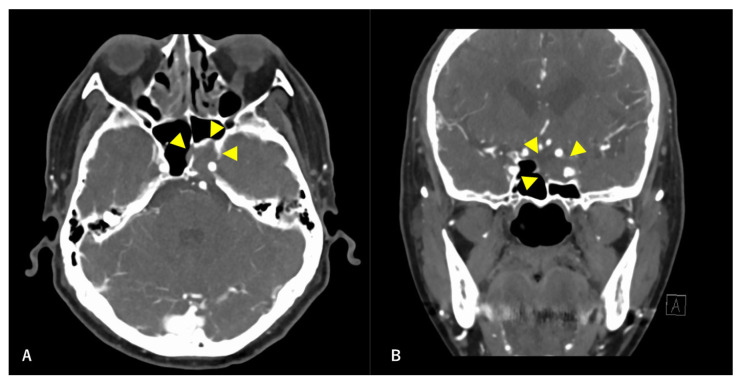
Preoperative contrast-enhanced computed tomography (CT) findings. Axial section (**A**) and coronal (**B**) images are shown. An isodense area with surrounding bone destruction is observed in the left sphenoid sinus, and no surrounding bone is observed around the internal carotid artery (ICA). Arrowheads indicate tumors.

**Figure 2:**
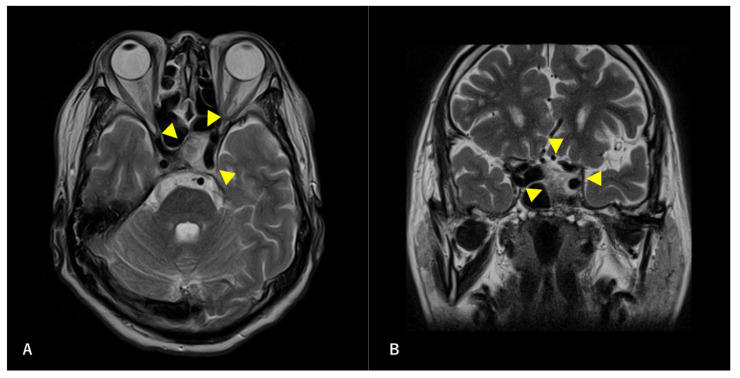
Preoperative T2-weighted magnetic resonance image (MRI) findings. Axial section (**A**) and coronal (**B**) images are shown. A high-intensity mass is observed in the left sphenoid sinus. The ICA is surrounded by a mass. Arrowheads indicate tumors.

**Figure 3:**
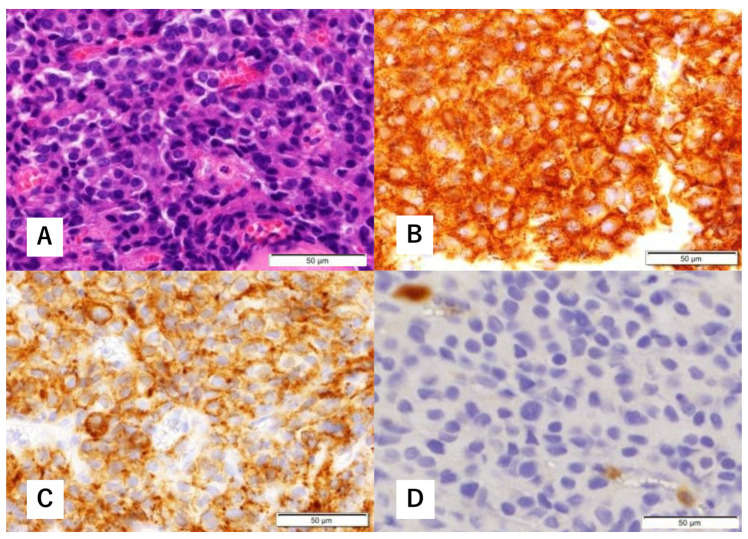
Histopathological findings of biopsy specimens. Hematoxylin-eosin (H&E) staining shows focal proliferation of atypical cells with a high nucleocytoplasmic (N/C) ratio (**A**). Immunohistological staining is positive for synaptophysin (**B**) and cluster of differentiation (CD) 56 (**C**). There are a few S-100 protein-positive cells (**D**).

A 60-year-old man without any symptoms presented to the otorhinolaryngology department with a suspected neoplastic lesion in the left sphenoid sinus, detected on magnetic resonance imaging performed during a physical examination. Imaging modalities showed that the tumor extended from the left sphenoid sinus to the area surrounding the ICA, with associated bone destruction, raising a suspicion of malignancy (Figure 1 and Figure 2). Neither regional lymph node nor distant metastases were observed. An otorhinolaryngologist performed a transnasal biopsy of the tumor under general anesthesia to determine the course of treatment. Immunohistochemical staining was positive for cluster of differentiation (CD) 56, synaptophysin, and chromogranin A. In contrast, cytokeratin AE1/AE3 staining was negative (Figure 3). Notably, there were few S-100 protein-positive cells, which are suggestive of the sustentacular cells typical of ONB. Because the diagnosis was difficult, the pathologists referred the specimens to a more specialized laboratory for additional testing. Further immunohistochemical tests were negative for both Nirenberg and Kim homeobox (NKX) 2.2 and CD99; therefore, the patient was diagnosed with olfactory neuroblastoma with Hymas pathological grade I and Kadish stage C.

**Figure 4:**
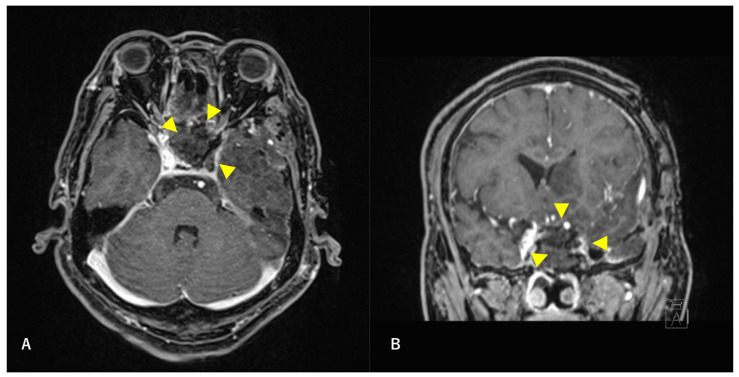
Postoperative T1-weighted contrast-enhanced MRI findings. Axial section (**A**) and coronal (**B**) images are shown. The tumor is confirmed to be resected along with the ICA. Arrowheads indicate the site of tumor removal.

**Figure 5:**
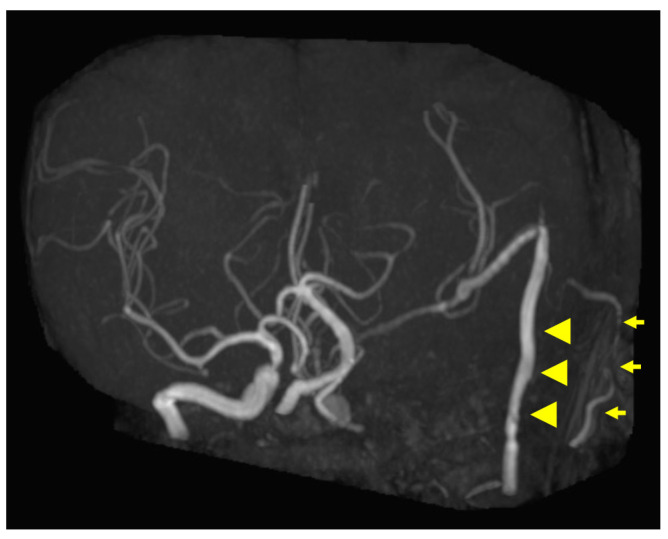
Postoperative magnetic resonance angiography (MRA) findings. MRA shows high-flow radial artery bypass between the M2 segment of the left middle cerebral artery (MCA) and the left cervical external carotid artery, with an additional superficial temporal artery (STA)–MCA bypass. Peripheral cerebral blood flow is maintained by the bypasses. The left internal carotid artery is trapped, and no blood flow is observed. Arrowheads indicate the high-flow bypass, and arrows indicate the STA–MCA bypass.

**Figure 6:**
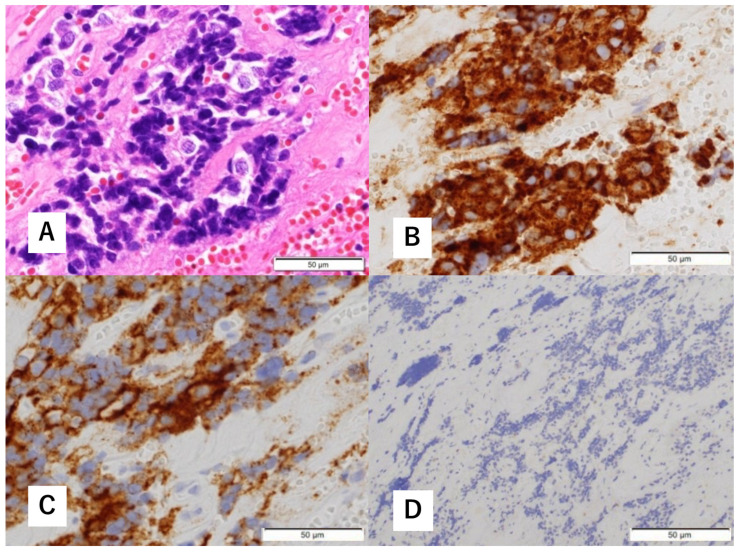
Histopathological findings of excised specimens. H&E staining shows a predominantly crushed artifact (**A**). Immunohistological staining is positive for synaptophysin (**B**) and CD56 (**C**). No S-100 protein-positive cells are observed (**D**).

A multidisciplinary team of otolaryngologists and neurosurgeons performed a complete resection of the tumor using a simultaneous combined transcranial and transnasal approach that utilized high-flow radial artery bypass between the M2 segment of the left middle cerebral artery (MCA) and the left cervical external carotid artery with an additional superficial temporal artery–MCA bypass. The margin of tumor resection was determined by a rapid intraoperative diagnosis to confirm the tumor extent. The ICA was trapped under blood flow preservation bypasses, enabling complete resection of the tumor along with the ICA (Figure 4). The ocular artery and cranial nerves III–VI were preserved. The skull base was reconstructed using two layers of the temporalis muscle and a vascular pedicle nasoseptal flap. Magnetic resonance angiography was performed immediately after the surgery, indicating adequate blood flow through the bypasses (Figure 5). The postoperative immunohistochemistry results were positive, similar to the biopsy immunoprofile (Figure 6). In addition, there were no S-100 protein-positive sustentacular cells around the tumor cells. Differentiating between high-grade ONB and neuroendocrine tumors is sometimes difficult; the tumor was eventually diagnosed as NEC, based on the absence of sustentacular cells and the absence of morphological findings such as Homer–Wright rosettes (Figure 6D).

Postoperatively, the patient had mild right-sided muscle weakness, transient expressive aphasia, and oculomotor nerve palsy, possibly due to surgery. A month after surgery, a lumboperitoneal shunt was added for postoperative hydrocephalus. Two months after surgery, the expressive aphasia improved, and the patient recovered to a modified Rankin scale (mRS) score of 4. Unexpectedly, the patient developed a traumatic acute subdural hematoma. Although an emergency craniotomy was performed to remove the hematoma, the mRS worsened to 5, and the patient became symptomatically fixed.

**Figure 7:**
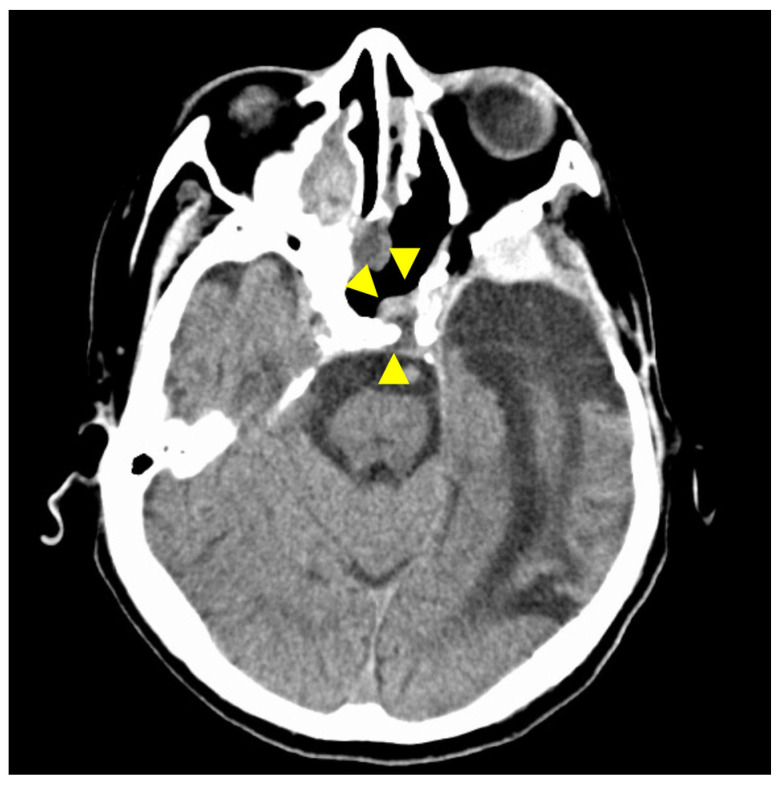
CT findings at 30 months after surgery. The isodense area at the tumor resection site shows no tendency to increase, and there is no evidence of recurrence on CT imaging. Arrowheads indicate the site of tumor removal. The isodensity in the left ethmoid sinus is due to chronic sinusitis.

Chemoradiation was planned for the postoperative period; however, considering the patient’s general condition, a decision was made not to apply additional treatment. No evidence of tumor recurrence on computed tomography was observed at 30 months after surgery (Figure 7).

## 3. Discussion

The clinical issues in this case were twofold. First, it should be noted that skull base malignancies are often diagnosed differently preoperatively and postoperatively. Second, although radical resection of a malignancy extending around the ICA is difficult, it can be achieved by utilizing high-flow bypass to preserve the blood flow.

A diagnosis of ONB based on preoperative biopsy often changes postoperatively [1,2], particularly for cases of ectopic ONB, which can be easily misdiagnosed as another tumor type [3]. Immunohistochemistry is important for accurate diagnosis; however, it can be difficult to make a diagnosis in cases in which neuroendocrine markers are positive and only a few S-100 protein-positive cells are present, as in this case. In this case, the S-100 protein-positive cells were determined to be sustentacular cells, and the diagnosis of ONB was made because other differential diseases were ruled out using additional immunohistochemical tests. Postoperative pathology showed no S-100 protein-positive cells in the resected specimen, suggesting that the S-100 protein-positive cells observed in the biopsied tissue may have been from residual normal tissue. Within the extent of our investigation, we found no reports of NEC misdiagnosed as ONB because of the presence of S-100-positive cells in biopsy tissue. It should be noted that S-100 protein-positive cells are also present in normal tissues (e.g., Langerhans and dendritic cells).

It is difficult to achieve complete resection of skull base tumors in cases that have invasion into or extension around the ICA, because vascular injuries are often life threatening, but complete surgical resection is preferable in cases in which the tumor is malignant. Revascularization with high-flow bypass allows trapping of the ICA and complete resection of the tumor [4,7]. Compared to other flow-preservation bypass procedures, high-flow bypass offers the advantage of maximal prevention of postoperative ischemic changes by the sufficient blood supply [5,8]. In this case, the preoperative diagnosis of ONB, for which the most effective treatment would be surgical removal that would require trapping of the ICA in conjunction with complete tumor resection, led to the choice of revascularization and tumor resection using high-flow bypass. Postoperative pathology tests revealed NEC; nevertheless, no tumor recurrence was observed because the surgical treatment involved radical resection, which can be effective regardless of the pathology. In addition, local ischemic changes were observed immediately after the surgery, but they did not worsen and tended to resolve over time with sufficient blood supply. If acute subdural hematoma had not occurred, the mRS score would have further recovered over time.

In this patient’s case, it is important to note that the misdiagnosis was due to not only the preoperative pathology but also to the clinical evaluation. Ectopic ONB occurs in various locations in the nasal sinuses [3,9,10], which is consistent with the presence of ONB in the sphenoid sinus; thus, both the pathologists and clinicians were convinced that the tumor was ONB. Ectopic ONB requires a critical assessment, since it often has a different definitive diagnosis. However, even if the final pathological diagnosis differs, a good clinical outcome can be expected if complete tumor resection is performed. Although advances in adjuvant therapy have limited the indications for complete tumor resection requiring high-flow bypass [5], complete tumor removal should be considered as a treatment strategy in situations in which the pathology is difficult to diagnose. On the other hand, our institution does not have a specialized oncologist to treat head and neck malignancies, and this may have biased our treatment strategy. Because only a limited number of institutions can perform revascularization using high-flow bypass, the best treatment option may vary depending on the characteristics of the institution.

## 4. Conclusions

A complete tumor resection utilizing high-flow bypass was performed for a NEC that was misdiagnosed preoperatively as an ONB. In cases in which malignant tumors extend around the ICA, complete resection of the tumor should be considered as a treatment strategy in situations when the preoperative pathology is not definitive.

## Data Availability

The data presented in this study are available on request from the corresponding author.
